# Comparative Cardiovascular and Renal Outcomes of Sodium-Glucose Cotransporter-2 Inhibitors and Glucagon-Like Peptide-1 Receptor Agonists in Type 2 Diabetes: A Systematic Review

**DOI:** 10.7759/cureus.80932

**Published:** 2025-03-21

**Authors:** Huzaifa K Khan, Zarbakhta Ashfaq, Hamza Jamil, Hureem Yaqoob, Nouman Anthony, Rimsha Dilawar, Bilal Ahmad, Sadikun Nabi, Sharmin Ahmed Rania, Falaknaz Saleem, Marie Gerges, Demiana M Mikhael

**Affiliations:** 1 Medicine, Foundation University School of Health Sciences, Islamabad, PAK; 2 Medicine, Rehman Medical Institute, Peshawar, PAK; 3 Oncology, Northwest General Hospital and Research Centre, Peshawar, PAK; 4 Emergency, Sahiwal International Hospital, Islamabad, PAK; 5 General Medicine, Rehman Medical Institute, Peshawar, PAK; 6 Community Medicine, Fauji Foundation Hospital Rawalpindi, Islamabad, PAK; 7 Medicine and Surgery, Foundation University Medical College, Islamabad, PAK; 8 Internal Medicine, Combined Military Hospital, Sialkot, PAK; 9 Radiology and Imaging, Bangladesh College of Physicians and Surgeons, Dhaka, BGD; 10 Radiology and Imaging, Combined Military Hospital, Dhaka, BGD; 11 Accident and Emergency, County Durham and Darlington NHS Foundation Trust, Durham, GBR; 12 Medicine, Surgery, Obstetrics and Gynecology, Dhaka Central International Medical College, Dhaka, BGD; 13 Public Health, University of Chester, Chester, GBR; 14 Oncology, Jinnah Postgraduate Medical Centre, Karachi, PAK; 15 Acute and Internal Medicine, George Eliot Hospital NHS Trust, Nuneaton, GBR; 16 Internal Medicine, Alexandria New Medical Center, Alexandria, EGY; 17 Emergency Medicine, Cairo University, Cairo, EGY

**Keywords:** cardiovascular outcomes, chronic kidney disease, combination therapy, glp-1 receptor agonists, heart failure, major adverse cardiovascular events, randomized controlled trials, renal outcomes, sglt2 inhibitors, type 2 diabetes

## Abstract

This systematic review evaluates the cardiovascular and renal outcomes associated with sodium-glucose cotransporter-2 (SGLT2) inhibitors and glucagon-like peptide-1 (GLP-1) receptor agonists in patients with type 2 diabetes, focusing on findings from randomized controlled trials. A comprehensive search was conducted across PubMed, Embase, and the Cochrane Library, adhering to the Preferred Reporting Items for Systematic Reviews and Meta-Analyses (PRISMA) guidelines. Seven high-quality trials met the inclusion criteria, with a combined focus on major adverse cardiovascular events (MACE), heart failure hospitalizations, all-cause and cardiovascular mortality, and renal function decline. SGLT2 inhibitors demonstrated superior efficacy in reducing heart failure and renal outcomes, while GLP-1 receptor agonists were more effective in preventing MACE and improving glycemic control. Combination therapy showed potential additive benefits, particularly in improving myocardial and arterial function. The findings highlight the complementary roles of these drug classes, underscoring the importance of personalized therapy based on individual patient profiles. Further research, including long-term head-to-head trials, is warranted to validate these results and optimize treatment strategies.

## Introduction and background

Cardiovascular disease (CVD) remains a leading cause of morbidity and mortality among individuals with type 2 diabetes mellitus (T2DM). Globally, over 32% of deaths in individuals with T2DM are attributed to CVD, with a significantly increased risk of atherosclerotic CVD (ASCVD) and heart failure due to chronic hyperglycemia, insulin resistance, and associated metabolic abnormalities [1,2]. A recent large-scale cohort study indicated that individuals with T2DM have approximately double the risk of cardiovascular events compared to those without diabetes, highlighting the urgent need for effective therapeutic interventions [3].

Recent pharmacological advancements have transformed the management of T2DM, with sodium-glucose cotransporter-2 (SGLT2) inhibitors and glucagon-like peptide-1 receptor agonists (GLP-1 RAs) becoming cornerstone therapies not only for glycemic control but also for cardiovascular risk reduction [4]. SGLT2 inhibitors work by inhibiting glucose reabsorption in the proximal renal tubules, leading to increased urinary glucose excretion, natriuresis, and subsequent reductions in intravascular volume and blood pressure. These mechanisms contribute to reduced preload and afterload, ultimately lowering the risk of heart failure and cardiovascular death [4]. GLP-1 RAs, on the other hand, act via the incretin pathway, enhancing glucose-dependent insulin secretion while suppressing glucagon release. Additionally, they exert anti-inflammatory and vasoprotective effects, improve endothelial function, and promote weight loss, collectively leading to a reduction in major adverse cardiovascular events (MACE), including myocardial infarction and stroke [5].

While both drug classes provide unique advantages, the comparative effectiveness of these therapies in mitigating cardiovascular risk in T2DM patients remains an area of active investigation. This systematic review synthesizes evidence from recent randomized controlled trials (RCTs) to determine the relative efficacy and safety of SGLT2 inhibitors versus GLP-1 RAs in reducing cardiovascular and renal outcomes in T2DM patients.

This systematic review follows the PICO (Population, Intervention, Comparison, and Outcome) framework to ensure a structured and evidence-based selection process [6]. The population of interest includes adults with T2DM who either have established cardiovascular disease or are at high risk for cardiovascular events. The intervention is treatment with SGLT2 inhibitors (e.g., dapagliflozin, empagliflozin, canagliflozin), while the comparison group comprises individuals treated with GLP-1 RAs (e.g., liraglutide, semaglutide, exenatide). The primary outcomes of interest include cardiovascular endpoints, such as MACE, hospitalization for heart failure, cardiovascular mortality, and renal function decline. Secondary outcomes encompass overall mortality, adverse events, and metabolic improvements. The PICO framework was systematically applied to guide database searches, define inclusion and exclusion criteria, and ensure the selection of studies with relevant patient populations and comparable treatment arms. This approach strengthens the validity and applicability of the findings in informing clinical decision-making and optimizing patient outcomes.

## Review

Materials and methods

Search Strategy

This systematic review adhered to the Preferred Reporting Items for Systematic Reviews and Meta-Analyses (PRISMA) guidelines [7] to ensure a transparent and standardized approach. A comprehensive search was conducted across PubMed, Embase, and the Cochrane Library to identify studies comparing the cardiovascular and renal outcomes of SGLT2 inhibitors and GLP-1 RAs in patients with type 2 diabetes. The search strategy included a combination of MeSH terms and keywords such as "Sodium-Glucose Cotransporter 2 Inhibitors," "SGLT2 inhibitors," "dapagliflozin," "empagliflozin," "Glucagon-Like Peptide-1 Receptor Agonists," "GLP-1 RAs," "semaglutide," "liraglutide," "cardiovascular outcomes," "renal outcomes," and "type 2 diabetes." Boolean operators (AND/OR) were used to combine search terms, and filters were applied to include only English-language articles and studies involving adult populations. The search encompassed articles published up to January 2025. Reference lists of included studies and relevant review articles were also screened to ensure a comprehensive retrieval of eligible studies. This structured approach ensured the identification of high-quality, relevant evidence to address the research question.

Eligibility Criteria

The eligibility criteria for this systematic review were defined to ensure the inclusion of high-quality evidence addressing the research question [8]. Only RCTs were considered, as they represent the highest level of evidence for evaluating the comparative effectiveness of SGLT2 inhibitors and GLP-1 RAs in patients with type 2 diabetes. Studies were included if they involved adult patients (aged 18 years or older) diagnosed with type 2 diabetes who were at high risk for cardiovascular or renal events or had established cardiovascular or chronic kidney disease. Eligible trials needed to compare SGLT2 inhibitors (e.g., dapagliflozin, empagliflozin) with GLP-1 RAs (e.g., semaglutide, liraglutide) or include either class compared to placebo, provided they reported cardiovascular or renal outcomes such as MACE, heart failure hospitalizations, cardiovascular death, or changes in renal function (e.g., eGFR decline).

Articles were limited to those published in English, with full-text availability and a focus on human subjects. Studies with a minimum follow-up period of 6 months were included to capture meaningful clinical outcomes. Trials that lacked sufficient data on cardiovascular or renal outcomes, those involving non-diabetic populations, or studies that primarily investigated non-clinical endpoints (e.g., laboratory biomarkers) were excluded. This rigorous set of criteria ensured the selection of trials with robust methodologies and clinically relevant findings to inform the comparative analysis.

Data Extraction

Data extraction was conducted systematically to ensure consistency and accuracy across all included studies. Two independent reviewers extracted data using a pre-designed data collection form, focusing on key study characteristics such as study design, sample size, population characteristics, intervention and comparator details, duration of follow-up, and primary and secondary outcomes. Extracted outcomes included MACE, heart failure hospitalizations, cardiovascular and all-cause mortality, and renal outcomes such as estimated glomerular filtration rate (eGFR) decline. Any discrepancies between reviewers were resolved through discussion or consultation with a third reviewer. Additionally, information on study methodology, risk of bias, and funding sources was collected to assess the quality and reliability of the included studies. This structured approach to data extraction ensured a comprehensive synthesis of findings relevant to the research question.

Data Analysis and Synthesis

The data analysis and synthesis were performed systematically to provide a comprehensive comparison of the cardiovascular and renal outcomes associated with SGLT2 inhibitors and GLP-1 RAs in type 2 diabetes. Quantitative findings from the included RCTs were synthesized descriptively due to the clinical heterogeneity in study populations, interventions, and reported outcomes. Key endpoints such as MACE, heart failure hospitalizations, cardiovascular death, and renal outcomes (e.g., eGFR decline) were compared across studies. Findings were organized thematically to reflect differences in drug efficacy based on specific patient subgroups, such as those with heart failure or chronic kidney disease. This approach allowed for a detailed narrative synthesis that emphasizes the strengths and limitations of each drug class while providing insights into their potential complementary roles in clinical practice.

Results

Study Selection Process

**Figure 1:**
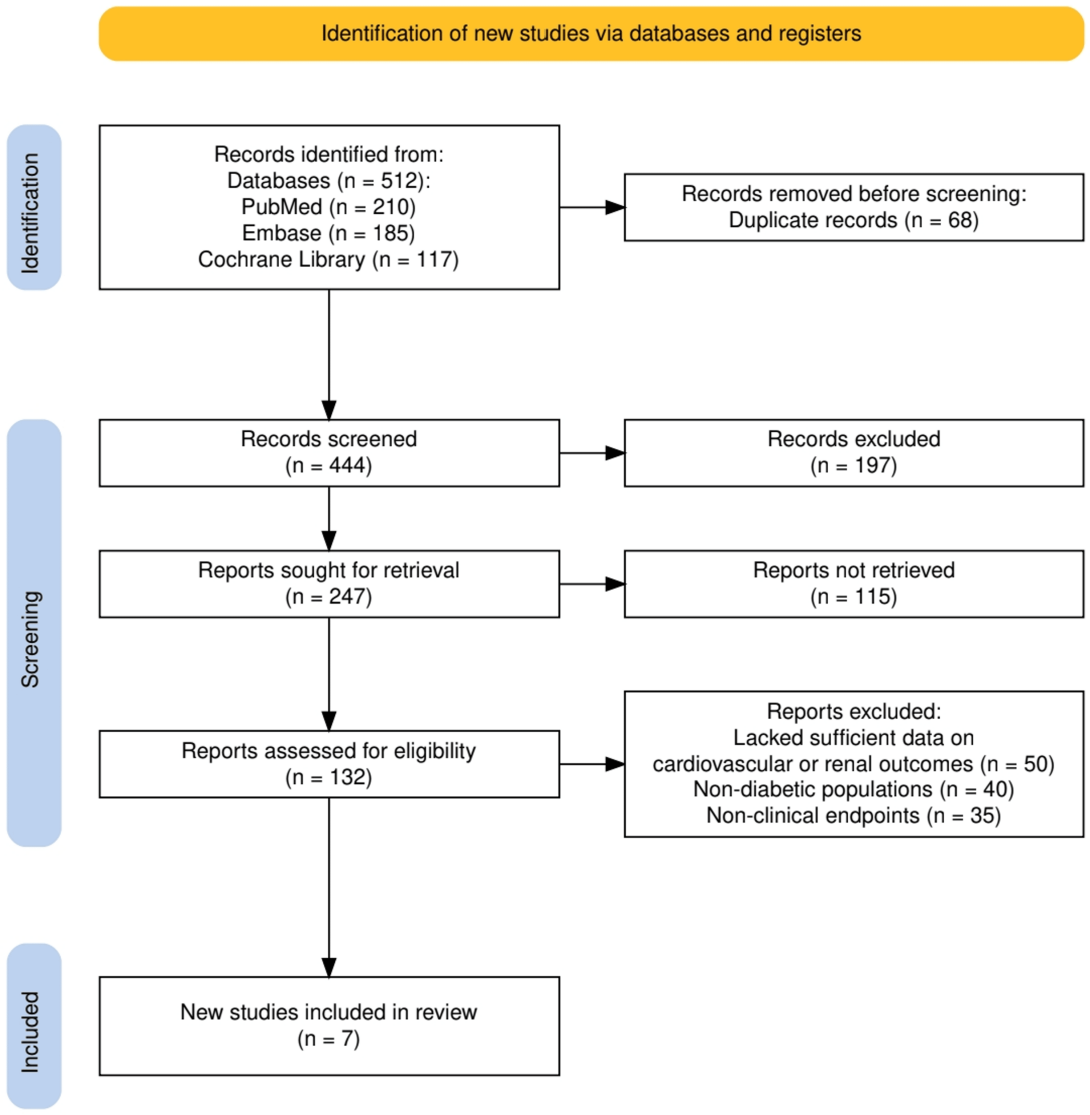
The PRISMA flowchart represents the study selection process. PRISMA: Preferred Reporting Items for Systematic Reviews and Meta-Analyses

The study selection process adhered to the PRISMA guidelines and is summarized in Figure 1. A total of 512 records were initially identified through database searches, including PubMed (210), Embase (185), and the Cochrane Library (117). After removing 68 duplicate records, 444 unique records were screened based on titles and abstracts. Of these, 197 records were excluded as they did not meet the predefined inclusion criteria. The remaining 247 reports were sought for full-text retrieval, but 115 could not be retrieved, leaving 132 reports for full-text assessment. After a thorough eligibility assessment, 125 reports were excluded due to various reasons: insufficient data on cardiovascular or renal outcomes (50), involvement of non-diabetic populations (40), or a focus on non-clinical endpoints (35). Ultimately, seven high-quality RCTs were included in the systematic review, providing robust evidence to address the research objectives. Figure 1 visually details the progression of study selection from identification to final inclusion.

Characteristics of the Selected Studies

The seven selected studies, summarized in Table 1, encompass RCTs and one posthoc analysis, all focusing on the cardiovascular and renal outcomes of SGLT2 inhibitors and GLP-1 RAs in patients with type 2 diabetes. The studies collectively included diverse populations, ranging from those with chronic kidney disease and cardiovascular disease to specific subgroups such as patients with severe insulin-deficient or insulin-resistant diabetes. Interventions primarily consisted of SGLT2 inhibitors such as dapagliflozin and empagliflozin or GLP-1 RAs such as semaglutide, liraglutide, and efpeglenatide, with comparators including placebo, insulin, or other pharmacological agents. The primary outcomes varied across trials but predominantly assessed MACE, heart failure hospitalizations, renal function decline (e.g., eGFR), and glycemic control. Key findings highlighted significant reductions in MACE, improved renal outcomes, and enhanced cardiovascular benefits, emphasizing the distinct strengths and complementary roles of these drug classes. The included studies demonstrated robust methodologies, ensuring high-quality evidence for this review.

**Table 1:** The summary of the included studies in the systematic review. T2DM: Type 2 Diabetes Mellitus; CKD: Chronic Kidney Disease; eGFR: Estimated Glomerular Filtration Rate; MACE: Major Adverse Cardiovascular Events; MI: Myocardial Infarction; CV: Cardiovascular; HR: Hazard Ratio; CI: Confidence Interval; GI: Gastrointestinal; SGLT2i: Sodium-Glucose Cotransporter 2 Inhibitors; GLP-1 RA: Glucagon-Like Peptide-1 Receptor Agonist; HbA1c: Glycated Hemoglobin; SIDD: Severe Insulin-Deficient Diabetes; SIRD: Severe Insulin-Resistant Diabetes; CVD: Cardiovascular Disease; PWV: Pulse Wave Velocity; AIx: Augmentation Index; BMI: Body Mass Index; ACM: All-Cause Mortality; HFrEF: Heart Failure with Reduced Ejection Fraction; HF: Heart Failure; KCCQ-TTS: Kansas City Cardiomyopathy Questionnaire Total Symptom Score

Study	Study Design	Population	Intervention	Comparison	Outcomes Measured	Key Findings
Gerstein et al., 2021 (AMPLITUDE-O) [9]	Randomized Controlled Trial	T2DM patients with a history of cardiovascular disease or CKD (eGFR 25–59.9 mL/min/1.73 m²) and ≥1 cardiovascular risk factor	Weekly subcutaneous efpeglenatide (4 or 6 mg)	Placebo	Primary: MACE (nonfatal MI, nonfatal stroke, CV death); Secondary: Renal outcomes (e.g., reduced eGFR, macroalbuminuria)	Efpeglenatide significantly reduced the risk of MACE (HR 0.73, 95% CI 0.58–0.92, P=0.007) and renal outcomes (HR 0.68, 95% CI 0.57–0.79, P<0.001). Common GI side effects included nausea, vomiting, and bloating.
Mann et al., 2024 (FLOW trial) [10]	Randomized Controlled Trial	T2DM patients with chronic kidney disease (CKD); stratified by SGLT2 inhibitor use at baseline (550 on SGLT2i, 2,983 not on SGLT2i)	Semaglutide (GLP-1 RA)	Placebo	Primary: Composite of kidney failure, ≥50% eGFR reduction, kidney death, CV death; Secondary: eGFR slope, MACE, all-cause death	Semaglutide reduced the risk of the primary composite outcome by 24% compared to placebo (HR 0.76; 95% CI 0.66–0.88, P < 0.001). Benefits were consistent across CV events and all-cause mortality, with no significant interaction based on SGLT2i use.
Dwibedi et al., 2024 [11]	Randomized Open-Label Trial	T2DM patients (n=239) with severe insulin-deficient diabetes (SIDD) or severe insulin-resistant diabetes (SIRD)	Semaglutide (GLP-1 RA)	Dapagliflozin (SGLT2i)	Primary: Change in glycated hemoglobin (HbA1c); Secondary: Fasting glucose, postprandial glucose, CV risk factors	Semaglutide reduced HbA1c more effectively than dapagliflozin (mean difference: -8.2 mmol/mol, 95% CI: -10.0 to -6.3). Greater reductions in fasting and postprandial glucose were observed in SIDD patients treated with semaglutide. Body mass index, blood pressure, and insulin resistance were useful predictors of glycemic and CV risk improvement.
Bechlioulis et al., 2023 [12]	Randomized Single-Blind Trial	T2DM patients (n=62, mean age 63 years, 25% with established CVD)	Liraglutide (GLP-1 RA), Empagliflozin (SGLT2i), and their sequential combination	Liraglutide vs. Empagliflozin (followed by combination therapy after 3 months)	Primary: Change in arterial stiffness indices (ΔPWV, ΔAIx); Secondary: Systemic vascular resistance, lipoprotein(a), body weight, HbA1c, blood pressure	No significant differences in arterial stiffness indices (PWV, AIx) between liraglutide, empagliflozin, or their combination. However, liraglutide showed greater improvements in systemic vascular resistance, BMI, visceral fat, blood pressure, and lipoprotein(a) levels.
Katogiannis et al., 2024 [13]	Randomized Controlled Trial	T2DM patients (n=200, mean age 59.5 years, 151 male) treated with metformin	Liraglutide (GLP-1 RA), Empagliflozin (SGLT2i), and their combination	Insulin-treated controls	Primary: Left atrial (LA) strain; Secondary: Arterial stiffness (PWV), central systolic blood pressure, myocardial function	Treatment with liraglutide, empagliflozin, and their combination improved left atrial reservoir strain and conduction strain compared to insulin. Combination therapy reduced PWV and central systolic BP more significantly than monotherapy with liraglutide or empagliflozin.
Clegg et al., 2019 (EXSCEL Analysis) [14]	Post Hoc Analysis of a Randomized Controlled Trial	Participants from the placebo arm of EXSCEL (n=1,418; 10.6% received SGLT2i, 5.2% received dapagliflozin)	SGLT2 inhibitors (e.g., dapagliflozin) administered as drop-in therapy	Non-SGLT2i users	MACE, all-cause mortality (ACM), estimated glomerular filtration rate (eGFR) slope	SGLT2i users had a numerically lower risk of MACE (HR: 0.79, 95% CI: 0.49–1.28) and significantly lower ACM risk (HR: 0.51, 95% CI: 0.27–0.95). Dapagliflozin users showed a slower decline in eGFR (+2.28 mL/min/1.73 m² per year, 95% CI: 1.01–3.54).
Butt et al., 2022 (DAPA-HF) [15]	Randomized Controlled Trial	Patients with heart failure with reduced ejection fraction (HFrEF) (n=4,744)	Dapagliflozin (SGLT2i)	Placebo	Primary: Composite of worsening HF or CV death; Secondary: HF hospitalization, all-cause mortality, CV death, KCCQ-TTS	Dapagliflozin significantly reduced the risk of worsening HF or CV death across all tertiles of the modified Heart Failure Collaboratory score (HR range: 0.71–0.76). Consistent benefits were observed for HF hospitalization, CV death, all-cause mortality, and quality of life (KCCQ-TTS).

Quality Assessment

The quality assessment of the included studies, as summarized in Table 2, demonstrated an overall low risk of bias across most domains, reflecting the robust methodologies employed in the selected trials. Randomization processes were clearly described, and the majority of studies ensured adequate allocation concealment and blinding, minimizing selection and performance biases. While the open-label design in one study introduced some concerns regarding measurement bias, all studies reported comprehensive outcome data with a low risk of reporting bias. The inclusion of high-quality RCTs and adherence to rigorous protocols provide confidence in the reliability of the synthesized findings.

**Table 2:** The quality assessment of the included articles.

Study	Randomization Process	Deviations from Intended Interventions	Missing Outcome Data	Measurement of Outcome	Selection of Reported Result	Overall Risk of Bias	Comments
Gerstein et al., 2021 (AMPLITUDE-O) [9]	Low Risk	Low Risk	Low Risk	Low Risk	Low Risk	Low Risk	Well-conducted RCT with robust methodology. No significant biases detected.
Mann et al., 2024 (FLOW trial) [10]	Low Risk	Low Risk	Low Risk	Low Risk	Low Risk	Low Risk	Randomization and stratification processes were clearly described. Comprehensive outcome reporting.
Dwibedi et al., 2024 [11]	Some Concerns	Low Risk	Low Risk	Low Risk	Some Concerns	Some Concerns	Open-label design introduces potential bias in measurement of subjective outcomes. Reporting of subgroup analyses lacked clarity in methodology.
Bechlioulis et al., 2023 [12]	Some Concerns	Low Risk	Low Risk	Low Risk	Low Risk	Some Concerns	Small sample size (n=62) and limited power for detecting differences in primary outcomes. Randomization process details were not fully reported.
Katogiannis et al., 2024 [13]	Low Risk	Low Risk	Low Risk	Low Risk	Low Risk	Low Risk	Well-conducted study with clear randomization, complete data reporting, and robust analysis methods.
Clegg et al., 2019 (EXSCEL Analysis) [14]	Some Concerns	Low Risk	Low Risk	Some Concerns	Some Concerns	Some Concerns	Post hoc analysis introduces inherent risks of bias. Propensity matching mitigates but does not eliminate confounding.
Butt et al., 2022 (DAPA-HF) [15]	Low Risk	Low Risk	Low Risk	Low Risk	Low Risk	Low Risk	Comprehensive trial design with adequate randomization, stratification, and rigorous reporting of outcomes.

Discussion

This systematic review synthesizes evidence from seven high-quality studies evaluating the cardiovascular and renal benefits of SGLT2 inhibitors and GLP-1 RAs in patients with type 2 diabetes. Overall, the findings underscore the efficacy of both drug classes in reducing cardiovascular and renal risks, though variations in study designs, patient populations, and follow-up durations may influence these outcomes. SGLT2 inhibitors, such as dapagliflozin and empagliflozin, were particularly effective in reducing the risk of heart failure and improving renal outcomes, as evidenced in the DAPA-HF [15] and EXSCEL [14] analyses, where reductions in worsening heart failure, cardiovascular death, and eGFR decline were significant. These benefits appeared more pronounced in patients with established heart failure and chronic kidney disease, while data in younger or lower-risk populations were less conclusive. Meanwhile, GLP-1 RAs, such as semaglutide and efpeglenatide, excelled in reducing MACE, including nonfatal myocardial infarction and stroke, as demonstrated in the AMPLITUDE-O [9] and FLOW [10] trials. Notably, some studies suggested greater cardiovascular benefits in men compared to women, although this requires further investigation. Differences in baseline characteristics between trials may also influence findings, as GLP-1 RAs were frequently studied in patients with atherosclerotic cardiovascular disease, whereas SGLT2 inhibitors showed greater efficacy in those with heart failure and chronic kidney disease. Despite these benefits, both drug classes were associated with side effects - SGLT2 inhibitors increased the risk of genitourinary infections and volume depletion, while GLP-1 receptor agonists frequently caused gastrointestinal symptoms such as nausea and vomiting. These findings highlight the need for personalized treatment approaches, considering both efficacy and tolerability based on individual patient profiles.

Notably, combination therapy of SGLT2 inhibitors and GLP-1 RAs showed additive benefits, particularly in improving myocardial and arterial function, as reported in studies by Katogiannis et al. [13] and Bechlioulis et al. [12]. While no significant differences in arterial stiffness indices were observed between monotherapy and combination therapy, GLP-1 RAs were more effective in reducing systemic vascular resistance, body mass index, and blood pressure. Additionally, findings from Dwibedi et al. [11] highlighted the superior glycemic control achieved with semaglutide compared to dapagliflozin, particularly in patients with severe insulin-deficient diabetes. Collectively, these results reinforce the need for personalized treatment strategies to optimize cardiovascular and renal outcomes in diverse patient populations with type 2 diabetes.

The findings of this systematic review are consistent with prior meta-analyses and large-scale trials, which have highlighted the cardiovascular and renal benefits of both SGLT2 inhibitors and GLP-1 RAs in type 2 diabetes [16]. Similar to the results reported in the EMPA-REG OUTCOME [17] and CANVAS [18] trials, SGLT2 inhibitors demonstrated significant reductions in heart failure hospitalization and renal disease progression across the included studies, with dapagliflozin showing a particularly robust effect in patients with heart failure and reduced ejection fraction (DAPA-HF). Similarly, GLP-1 RAs, as seen in the LEADER [19] and SUSTAIN-6 [20] trials, effectively reduced MACE, consistent with the findings of the AMPLITUDE-O [9] and FLOW [10] trials in this review. These results align with the current recommendations of the American Diabetes Association (ADA) and the European Society of Cardiology (ESC), which endorse these therapies for high-risk cardiovascular and renal patients with type 2 diabetes.

However, discrepancies with prior research were also noted, particularly in the findings on combination therapy. While this review found additive benefits in improving arterial and myocardial function when combining GLP-1 RAs and SGLT2 inhibitors, earlier studies have shown mixed results, with some trials reporting no significant advantage of combination therapy over monotherapy. Variations in study designs, including differences in patient populations, baseline risk factors, and the duration of follow-up, may account for these inconsistencies. For instance, studies focusing on younger, healthier populations may underestimate the cardiovascular benefits observed in higher-risk cohorts. Furthermore, differences in endpoints, such as prioritizing glycemic control over cardiovascular outcomes, may contribute to variability in reported findings [21]. These insights highlight the importance of tailoring therapy based on individual patient profiles and underscore the need for future research to clarify the long-term effects of combination strategies.

The observed cardiovascular and renal benefits of SGLT2 inhibitors and GLP-1 RAs can be attributed to their distinct yet complementary mechanisms of action. SGLT2 inhibitors, such as dapagliflozin and empagliflozin, reduce renal glucose reabsorption, leading to glycosuria and improved glycemic control. Additionally, their natriuretic and osmotic diuretic effects lower preload and afterload, thereby reducing the risk of heart failure and slowing renal disease progression [22]. These benefits are further augmented by their ability to decrease systemic blood pressure and mitigate oxidative stress. Conversely, GLP-1 RAs, including semaglutide and liraglutide, improve glycemic control through enhanced glucose-dependent insulin secretion and suppression of glucagon release. Their anti-inflammatory and anti-atherosclerotic properties, combined with significant reductions in body weight and improvement in endothelial function, likely explain their robust effects on MACE [23]. Together, these mechanisms support the observed superiority of SGLT2 inhibitors in heart failure-related outcomes and GLP-1 RAs in atherosclerotic cardiovascular disease prevention while highlighting the potential for synergistic benefits when used in combination.

This systematic review stands out for its robust methodology, which includes the comprehensive synthesis of recent high-quality RCTs and post hoc analyses. By incorporating studies such as the AMPLITUDE-O [9], FLOW [10], and DAPA-HF [15] trials, this review provides a detailed and contemporary comparison of SGLT2 inhibitors and GLP-1 RAs, highlighting their distinct strengths and complementary roles in cardiovascular and renal protection. Furthermore, the inclusion of trials evaluating combination therapy addresses a critical research gap, offering new insights into the potential additive benefits of these pharmacological agents. These findings contribute to clinical practice by guiding personalized therapy selection for type 2 diabetes patients at high cardiovascular and renal risk, emphasizing tailored strategies for heart failure versus atherosclerotic cardiovascular disease prevention.

Despite its strengths, this review is not without limitations. The heterogeneity of the included studies, particularly in terms of patient populations, baseline characteristics, and endpoints, may introduce variability in the pooled findings. Additionally, the lack of direct head-to-head trials between SGLT2 inhibitors and GLP-1 RAs limits the ability to draw definitive conclusions about their comparative effectiveness. Many studies also had relatively short follow-up durations, which may underestimate long-term outcomes such as sustained renal protection or cardiovascular mortality benefits. Potential publication bias, inherent to systematic reviews, could further influence the results. These limitations underscore the need for more focused, long-term head-to-head trials to validate the observed findings and refine therapeutic recommendations.

The findings of this systematic review provide valuable guidance for optimizing treatment decisions in patients with type 2 diabetes and cardiovascular or renal risks. SGLT2 inhibitors, such as dapagliflozin, are particularly well-suited for patients with heart failure, given their ability to reduce hospitalizations and cardiovascular mortality through mechanisms such as natriuresis and hemodynamic stabilization. Conversely, GLP-1 receptor agonists, such as semaglutide and liraglutide, are more effective in reducing MACE, including myocardial infarction and stroke, making them ideal for patients with atherosclerotic cardiovascular disease or those at high risk for such events. The potential additive benefits of combination therapy in improving arterial function and glycemic control further highlight its role in select patient populations. These insights emphasize the importance of individualized therapy based on a patient’s comorbidities and risk profile, aligning with guideline-directed recommendations to improve long-term outcomes in this vulnerable population.

Future research should address several critical gaps identified in this systematic review. Long-term studies are needed to evaluate the sustained efficacy and safety of SGLT2 inhibitors and GLP-1 RAs, particularly in diverse and underrepresented subpopulations, such as patients with advanced chronic kidney disease or those at high cardiovascular risk with limited comorbidities. Direct head-to-head trials comparing these drug classes would provide definitive insights into their relative effectiveness and help refine therapeutic algorithms. Additionally, the long-term benefits and potential synergistic effects of combination therapy require further investigation to determine whether such strategies can optimize cardiovascular and renal outcomes. Finally, cost-effectiveness analyses could play a vital role in guiding clinical decisions and healthcare policies, ensuring that these therapies are accessible and economically sustainable for broader patient populations [24]. Addressing these research gaps will enhance the evidence base and support personalized, value-based care in type 2 diabetes management.

## Conclusions

Both SGLT2 inhibitors and GLP-1 RAs provide substantial cardiovascular and renal benefits for patients with type 2 diabetes, with each drug class demonstrating unique strengths. SGLT2 inhibitors are particularly effective in reducing heart failure-related outcomes and slowing renal disease progression, while GLP-1 RAs excel in reducing atherosclerotic cardiovascular events, such as myocardial infarction and stroke. The potential for combination therapy to offer additive benefits further highlights the importance of personalized treatment strategies tailored to the specific comorbidities and risk profiles of individual patients. This review reinforces the value of these therapies in improving long-term outcomes and emphasizes the need for future research to address remaining gaps and optimize therapeutic approaches.
